# Role of delayed salvage bevacizumab at symptomatic progression of chemorefractory glioblastoma

**DOI:** 10.1186/s12885-019-5678-1

**Published:** 2019-05-14

**Authors:** Moire Cuncannon, Matthew Wong, Dasantha Jayamanne, Linxin Guo, Nicola Cove, Helen Wheeler, Michael Back

**Affiliations:** 1 Central Coast Cancer Centre, Gosford Hospital, Gosford, NSW 2250 Australia; 2 Northern Sydney Cancer Centre, Royal North Shore Hospital, Reserve Rd, St Leonards, NSW 2065 Australia; 3 Sydney Neuro-Oncology Group, Sydney, NSW Australia; 4 Sydney Medical School, University of Sydney, Camperdown, NSW 2006 Australia

**Keywords:** Glioblastoma, Bevacizumab, Salvage, Survival

## Abstract

**Background:**

Assess benefit of salvage bevacizumab (BEV) at time of symptomatic progression in patients with refractory glioblastoma (GBM).

**Methods:**

Patients managed with adjuvant long course chemo-radiation therapy for GBM were entered into a prospective database. At chemorefractory symptomatic progression, patients were offered BEV or best supportive care. Re-irradiation (ReRT) was used with BEV in selected patients. BEV continued indefinitely until deterioration limited hospital based infusion.

The primary endpoint was median survival calculated from date of decision for BEV to proceed (BEVstart), or decision to decline BEV (BEVreject).

**Results:**

Fifty-five patients were managed of which 48 patients have relapsed disease. The median survival post relapse was 6 months (95%CI: 4.6–7.4). At relapse, 28 patients received BEV with only 14% delivered at first relapse. The median number of BEV cycles was 8 (range 1–25). ReRT was subsequently used in 16 (33%) relapsed patients. BEV treated patients were associated with improved median survival post relapse with 9 months vs 3 months (*p* < 0.01).

The median survival from BEV related decision-making at symptomatic refractory progression to death was 4 months (95%CI: 2.0–6.0). BEVstart was associated with improved survival from this date with median survival of 6 months vs 1 month with BEVreject (p < 0.01). Median survival with ReRT from this date was 8 months vs 3 months without ReRT (*p* = 0.01). In the BEV patients at eventual progression, death occurred at a median of 30 days post BEV cessation.

**Conclusion:**

In this clinic managing selected patients with chemorefractory progressive glioblastoma, delayed salvage bevacizumab, often in combination with re-irradiation, may provide an increase in survival duration compared with best supportive care.

## Background

Glioblastoma (GBM) is a highly malignant primary brain tumour in adults with a poor prognosis involving a median survival of 17 months [1, 2]. These tumours are highly angiogenic with elevated levels of vascular endothelial growth factor (VEGF) however two large randomised trials have failed to demonstrate an overall survival benefit of VEGF blockade by Bevacizumab (BEV) in the adjuvant therapy of patients with newly diagnosed GBM [3, 4]. However, at time of recurrent disease there is increased utilisation of BEV because of potential benefits as a steroid substation agent to reduce intracranial pressure. Four clinical trials have shown a survival beyond 6 months in patients managed with BEV at relapse, either as monotherapy [5, 6] or with a systemic chemotherapy agent [7, 8]; but the timing, need for combination therapy, and magnitude of benefit remains uncertain. Despite the United States FDA listing approval for BEV in recurrent disease in December 2017 [9], there is a paucity of data regarding magnitude of benefit over best supportive care [5]. This is in the presence of a therapy which may have significant financial cost as well as exposing patients to risk of morbidity.

This current study undertaken in a regional cancer centre explores a policy in the care of patients with recurrent GBM of delaying monotherapy BEV until time of symptomatic chemorecfratory disease at either second or later relapse after salvage systemic therapy [10].

## Methods

Consecutive patients managed at a regional cancer center, with adjuvant chemo-radiation therapy as per the EORTC-NCIC Protocol [2] for newly diagnosed GBM from March 2013 to December 2016 were entered into a prospective database, approved by an Institutional Ethics Review Board. Elderly patients managed with hypofractionated RT were not included in the analysis. No patients were enrolled onto an intercurrent clinical trial during this study period. All patients were followed clinically until death or the censure date of the study on 1st February 2018.

### Management of Relapse

Patients were reimaged with MRI 1 month after the 6 weeks concurrent chemo-radiation; then every 2 months until completion of adjuvant TMZ. Then MRI scans were performed 3 monthly for 2 years and then 4 monthly; or earlier at the time of change in symptoms. Progression was determined based on the Revised Assessment in Neuro-Oncology (RANO) criteria [11]. Any late RT effects were identified by correlating change in sequential MRI findings with RT treatment fields. In these patients a repeat MRI was performed initially 1 month later, then either monthly or second monthly until resolution or progression of gadolinium enhanced T1 lesions.

Once radiological progression was confirmed the date of initial relapse was recorded. Salvage therapy involved either a surgical debulking followed by second line systemic chemotherapy; or second line systemic chemotherapy alone. The salvage regimen utilised depended on the timing of progression. If later relapse beyond 4 months from cessation of adjuvant phase, then there was recommencement of temozolomide with addition of procarbazine; or if earlier relapse then lomustine/procarbazine. If not utilised at first relapse, then lomustine was introduced at second line. BEV was not utilised at first relapse unless there was significant steroid refractory raised intracranial pressure or persistent bone marrow suppression contraindicated the use of salvage chemotherapy.

**Fig. 1:**
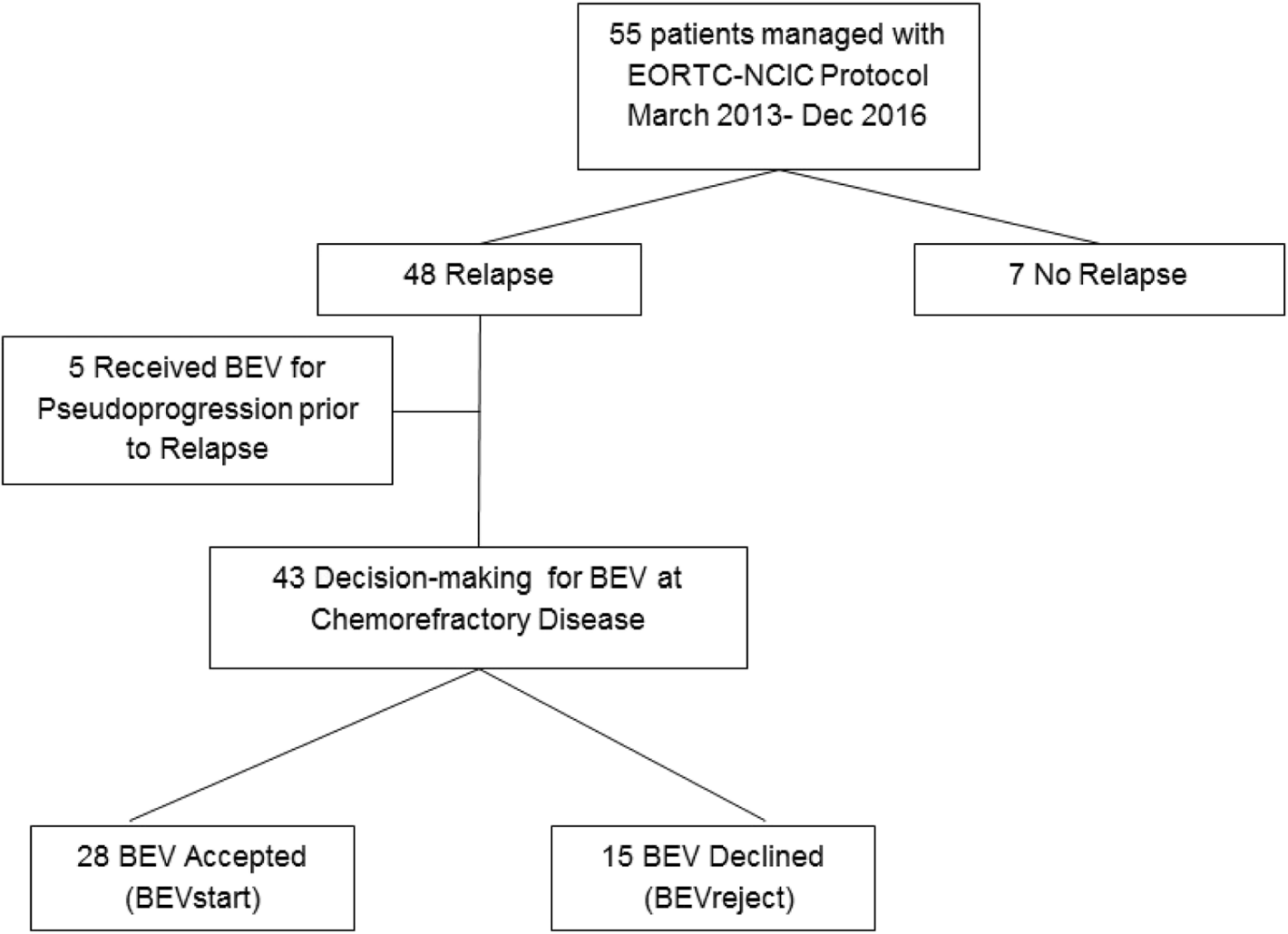
Flowchart of patients leading to decision-making for bevacizumab (BEV) at time of chemorefractory relapse

Chemorefractory symptomatic progression was defined as increased symptoms requiring dexamethasone, and enlarging contrast enhancing mass following at least one regimen of salvage chemotherapy. At this stage a decision-making process was undertaken with the patient by both medical and radiation oncology teams regarding further intervention with BEV or best supportive care (BSC). This was recorded as the date of BEV decision (Fig. 1), and BEV was commenced in the following 5 days. Use of BEV may allow later offer of re-irradiation (ReRT) depending upon pattern of relapsed disease [12, 13]. BEV was administered at 10 mg/kg intravenously every 2 weeks; and continued indefinitely until deterioration of performance status limited hospital based infusion. BEV required patient funding under a pharmaceutical access scheme involving a patient co-payment to a maximum of approximately 12,000 EURO.

Response to BEV was followed on MRI with DWI sequences assisting in determining outcome with first assessment being at 1 month post initiation. The use of large volume fractionated re-irradiation (ReRT) was individualised and highly selected. It was utilised only in patients who were on BEV [13], and the timing varied between planned immediate ReRT at time of BEV initiation (generally after 2 infusions and response assessment) or at time of subsequent BEV refractory disease noted by MRI progression. The ReRT regimen involved intensity modulated radiation therapy delivered at a dose of 35-40Gy in 15 fractions over 3 weeks, accepting overlap regions exceeding 100Gy. [13].

### Data collection

Data collected included initial prognostic factors and treatment details; date of initial diagnosis; BEV decision and date of last follow-up or death; BEV dates and cycles; ReRT use; dexamethasone dose; ECOG status; neurocognitive function; and dates of radiological progression.

### Endpoints

The primary endpoint was the median survival post the decision-making for BEV at time of chemorefractory relapse. This was measured from the time-point where the offer to utilise BEV was provided to the patient; and a decision was either to proceed with BEV (BEVstart), or decision to decline BEV (BEVreject). Secondary survival endpoints included overall survival (OS) and progression-free survival (PFS) calculated from date of initial diagnosis; as well as survival post first (BEVstart), and last cycle (BEVend) of bevacizumab. Clinical endpoints included dexamethasone dose and functional status 1 month post first BEVstart.

### Statistical methods

The Kaplan Meier technique was used to estimate and plot survival for endpoints (overall survival, relapse free survival and survival post-relapse). For key patient and disease characteristics, log-rank tests were used to identify predictive variables. Estimates of the effects of these groups is computed using Cox proportional hazards regression (presented with HR and 95%CI).

## Results

Fifty-five patients were managed under the EORTC-NCIC protocol [9] during the study period. Initial patient and tumour characteristics are detailed in Table 1. Twelve patients (22%) were aged less than 50 years. Only one patient had a GBM with IDH mutation; remaining progression free at 15 months and thus not included in the relapse analysis. Twenty-eight patients (51%) had a near-total resection and forty-three (78%) had ECOG 0,1 status at start of RT. MGMT methylation status was only available on 51% of patients, and of these 43% had methylated tumours. Forty-five patients have died with a median follow-up of 17.5 months in ten remaining survivors at data censure. One patient death was unrelated to disease progression and was removed from analysis regarding BEV decision. Median OS from date of initial diagnosis was 17.0 months (95%CI: 14.8–19.2).

**Table 1:** Patient and Tumour Characteristics at initial diagnosis

Subgroup	Total Group(55)	BEVstart(28)	BEVreject(15)
Age			
< 50	12	7	3
> 50	43	21	12
Gender			
Male	31	12	13
Female	24	16	2
Site of Tumour			
Frontal	18	10	6
Temporal	13	8	3
Parietal	19	7	5
Occipital	3	3	0
Other	2	0	1
Extent of Resection			
Near Total	28	15	6
Subtotal	18	9	7
Biopsy	9	4	2
ECOG at initial RT			
0,1	43	22	11
2,3	12	6	4
MGMT			
Methylated	12	9	2
Unmethylated	16	4	2
Unknown	27	15	11

Forty-eight patients have relapsed disease with median PFS being 11.0 months (95%CI: 9.0–13.0). The median survival post relapse was 6 months (95%CI: 4.6–7.4); and 4 patients were alive at time of censure. Salvage therapy at time of first relapse is listed in Table 2. Eighteen patients had repeat craniotomy, 45 patients received salvage chemotherapy, which included more than one salvage regimen in 42 patients. ReRT was used in 16 (33%) of patients with relapse, and was delivered with BEV in all 16. BEV was utilised in 33 patients of which 5 received treatment prior to relapse to manage pseudoprogression events (Fig. 1). Fifteen patients declined BEV at relapse, predominantly on concerns over financial cost. Survival outcome in relation to total BEV use is described in Table 3. Patients who subsequently received BEV had a median survival from date of initial relapse of 9 months (95%CI: 6.0–12.0). This compared with 3 months (95%CI: 0.9–5.1) in those not receiving BEV (*p* < 0.01).

**Table 2:** Details of Salvage therapy

Subgroup	Number (55)
Relapse	
Nil	7
Yes	48
Repeat Craniotomy	
No relapse	7
Craniotomy	18
Nil	30
Re-irradiation	
No relapse	7
ReRT	16
Nil	32
BEV use	
No relapse	7
Pseudoprogression	5
Relapse	28
Nil	15
Timing of BEV at Relapse	(n=28)
First relapse	4
Second relapse	8
Third relapse	16
Number of BEV cycles at Relapse	
Median	8
Range	1-25

**Table 3:** Median Survival Outcomes with BEV

	Median Survival	95% CI (55)
Overall Survival from Initial Diagnosis (*n* = 55)	17.0 months	14.8–19.2 months
Progression Free Survival from Initial Diagnosis (n = 55)	11.0 months	9.0–13.0 months
Overall survival from relapse (*n* = 48)	6.0 months	4.6–7.4 months
Overall Survival from BEV decision-making (*n* = 43)	4.0 months	1.0–7.2 months
Overall Survival from BEV decision-making with BEVstart (n = 28)	6 months	4.4–7.6 months
Survival from BEV cessation (*n* = 28)	30 days	7–65 days

For the BEV decision analysis, the 5 patients managed previously for pseudoprogression were excluded, even though in those patients the BEV continued to be delivered during subsequent relapse care (Fig. 1). The remaining 28 patients received BEV at relapse with 24 (86%) patients treated at second or later relapse following 1–3 courses of salvage chemotherapy. The four (14%) patients that were managed at first relapse either declined subsequent chemotherapy (2 patients) or had marrow suppression limiting salvage chemotherapy (2 patients). The median number of BEV cycles was 8 (range 1–25).

**Fig. 2:**
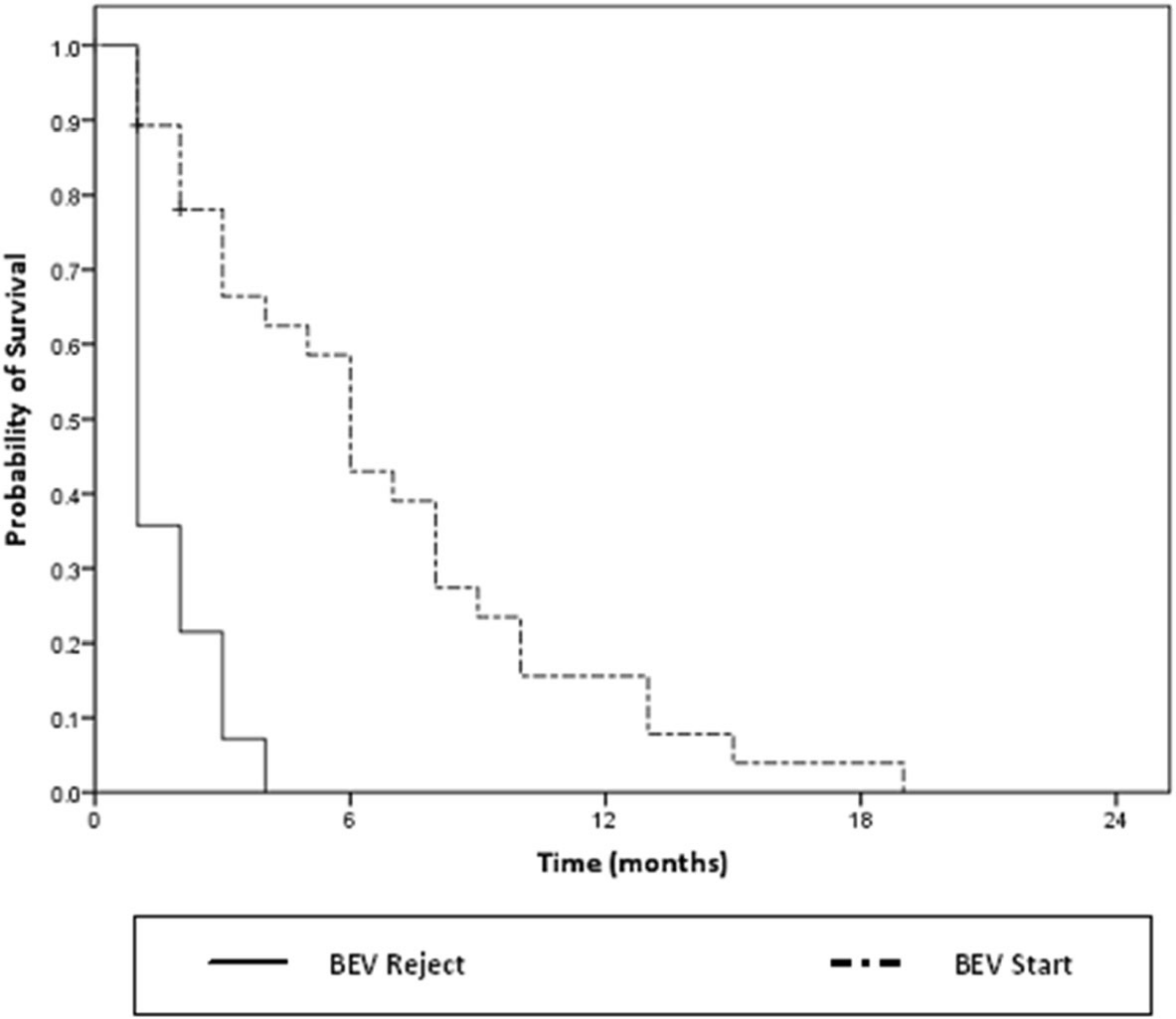
Survival from date of BEV decision-making for patients with BEVstart (light) and BEVreject (dark)

The median survival from the BEV related decision-making (BEVstart or BEVreject) in the 43 eligible patients with symptomatic refractory progression to death was 4.0 months (95%CI: 1.0–7.2). BEV was associated with improved survival from this date (Fig. 2) with median survival of 6 months (95%CI: 4.4–7.6) vs 1 month with BEVreject (*p* < 0.01). Analysis was performed excluding the 4 patients who were managed with BEV at first relapse, and there was no alteration to the median survival of 6 months.

Median survival with ReRT from date of BEV related decision-making was 8 months vs 3 months without ReRT (p < 0.01). To clarify the relative contribution of ReRT, a further hypothesis generating analysis was performed on the BEVstart patients and outcome based on use of ReRT. Understanding that patients did not start ReRT until 2 months after the initiation of BEV, the 16 BEVstart patients who received ReRT and the 9 (out of 12) BEVstart patients who were alive at 2 months and did not receive ReRT were analysed. The median survivals were 8 months and 6 months respectively and did not reach statistical significance.

Baseline patient and tumour characteristics were analysed as to the association of factors with survival post decision-making. As above, the use of BEV (BEVstart) and ReRT were associated with longer median survival. MGMT methylation did reach significance (*p* = 0.02) however strength of this association would be limited by the small number of patients (39% of patients in the analysis) for whom this characteristic was available. Patient age (*p* = 0.41), Initial ECOG (*p* = 0.73), Extent of resection (*p* = 0.31) and Repeat craniotomy (*p* = 0.33) were not associated with survival post BEV decision-making. There was no factor, including the limited MGMT methylation data, which independently predicted for survival on multivariate analysis.

**Fig. 3:**
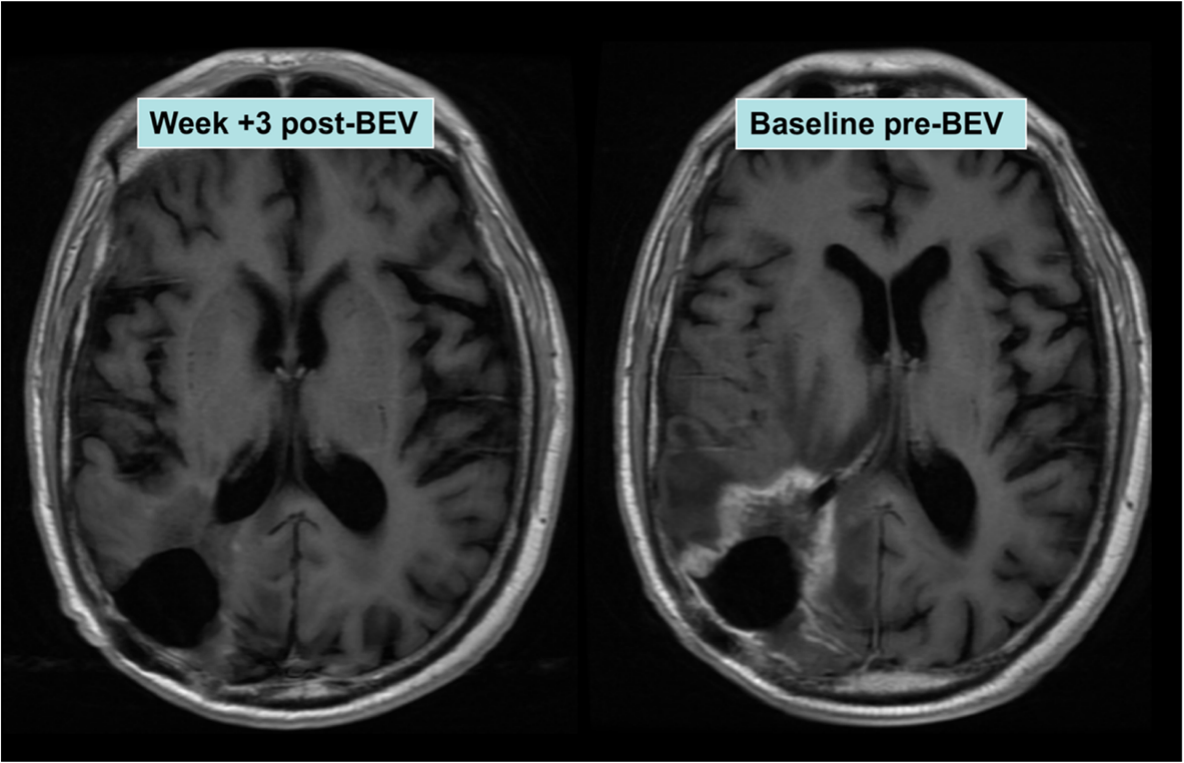
Example of radiological response to bevacizumab (BEV)

At 1 month post BEVstart, all patients had a reduction in dexamethasone dose by at least 50% or to 0.5 mg. A radiological response was confirmed in all patients with a repeat MRI (Fig. 3). In no patient was BEV ceased due to toxicity, and cessation of BEV occurred when patients’ performance status limited further outpatient therapy. In these BEV patients at eventual progression, death occurred at a median of 30.5 days after last dose of BEV.

## Discussion

This study demonstrates that salvage BEV at time of chemorefractory disease for GBM may provide a median 5 months additional survival and a 25% chance of being alive at 9 months post commencement. This compares with best supportive care at the same period which resulted in deterioration and a median survival of 4 weeks. Most importantly in this study population, the use of BEV allowed large volume re-irradiation (ReRT) to be offered to selected patients and that may be an important factor in the extension of survival duration. Importantly the quality of survival is good with stable or improved function reflected by steroid dose minimisation.

Although this study has inherent potential bias given the non-randomised nature of the treatment allocation and decision based on patient choice, it is hypothesis generating that in selected patients delaying BEV to late salvage at time of chemorefractory disease may provide an extension of survival beyond best supportive care alone. The historical randomised clinical trials exploring BEV with relapsed GBM have had conflicting outcomes, but in these trials the BEV was administered predominantly at initial relapse. Randomised studies BRAIN [5] and CABARET [6] examined BEV either as single agent or in combination with chemotherapy and showed varying results. A positive outcome in median survival extension with irinotecan and BEV in BRAIN but no benefit with carboplatin in CABARET. The median survival in the BEV alone arms were 8.7 months and 7.5 months respectively. BELOB randomized Phase II compared BEV and lomustine to BEV or Lomustine alone [8] at first relapse. It showed no benefit for BEV alone over lomustine-BEV combination with a median survival in the BEV alone arm of 8 months [8]. In the current report the median survival with BEV alone was 6 months, but notably this is in chemorefractory disease where 84% of patients were managed at second or third relapse. This differs from the three clinical trials which were generally at first relapse, with the rates of patients being managed at second or later relapse being 18, 33 and 0% [5, 6, 8]. Additionally, the recently reported EORTC 26101 BELOB Phase III trial [14] also randomised patients at first progression, with 34% of patients having excellent performance status and 51% not requiring corticosteroids at baseline. This showed no benefit of combination BEV-lomustine over lomustine alone, thus suggesting that in the absence of raised intracranial pressure symptoms or chemorefractory disease an initial approach exploring lomustine alone could be attempted prior to salvage with BEV.

The availability of MRI scanning for assessment of chemotherapy response has meant that earlier diagnosis of refractory disease can be detected in the presence of minimal symptoms. Different to other malignancies where progressive systemic disease results in symptoms such as lethargy, pain, anorexia and other generalised effects, the symptoms from progressive intracranial disease may be minimal or focal, and corticosteroids leads to potential partial response. If tumour is distant to motor tracts, then both mobility and functional status may be maintained. Radiological progression may then be an advance predictor for functional decline from raised intracranial pressure and patients may be seeking an additional palliative therapy to delay this event. Unfortunately corticosteroids may already be utilised with associated dose dependent morbidity of weight gain, proximal myopathy, or mood changes which may be the major symptoms for patients. This is a limiting factor in further corticosteroid dose escalation with presence of new symptoms.

BEV through inhibition of tumour VEGF production may produce an effective yet temporary steroid sparing effect that is clinically meaningful to patients through the avoidance of steroid morbidity [15]. Logistically, it has minimal impact on patients as the toxicity is generally dose duration related and stochastic effects such as intracranial bleed and venous thrombosis are low and clinically acceptable [16]. In the current patient cohort, the limitation was generally financial with no government support and a significant patient co-payment required [12]. Planning for intervention involved open patient discussion with multidisciplinary team regarding limitation of the therapy given, issues of poor wound healing restricting further neurosurgical procedures, and principles of BEV cessation when infiltrative disease leads to worsening mobility and performance status.

In recurrent GBM, the timing of BEV remains uncertain [5]. In this study, BEV was delivered at time of chemorefractory disease and following two salvage therapies in 58% of patients. For this patient group the clinical aim is palliative and outcome likely temporary. Understanding that palliative situation, and an associated potential financial cost, then delaying BEV to a point of radiologically refractory disease may be more appropriate. The decision also needs to be considered in regard to steroid requirements and intervening before steroid refractory disease that has led to related morbidity. Clinical trials such as CABARET, BRAIN and BELOB which showed a limited benefit to BEV utilised it at first relapse [5–7]. This may mean the symptomatic clinical benefit is diluted amongst patients who have the potential for other options or at a point where threatening symptomatic raised intracranial pressure is not immediately present. In the current study patients were closely monitored by both radiation and systemic therapy teams with clinical assessment and radiological monitoring every 2–3 months. Thus, the aim was to intervene early once chemorefractory, and before steroid morbidity or other neurological deficits arose. It was an active policy in preparing patients for BEV decision-making when clinically appropriate.

The role of ReRT is not well defined and most ReRT data in the literature involves small volume hypofractionated therapies delivered at early relapse and generally without the clinical need for BEV [17]. This study delivered ReRT late and thus to large volumes of contrast enhancing recurrent disease not suitable for short hypofractionated RT regimens or stereotactic radiosurgery [13, 18]. The treatment can be tolerated well because any acute inflammatory or necrotic events are minimised by the concurrent BEV [13, 17, 19, 20]. For these reasons, only patients receiving BEV were offered ReRT and it was only utilised in half of these patients. The selection criteria were poorly defined and likely bias the patients with improved performance status. By the nature of a patient co-payment requirement to receive BEV, demonstrates an active involvement and commitment to optimising outcomes of the regimen; as well as a likely presence of a higher socioeconomic status and support network. Indeed experiencing an initial response to BEV is more likely to encourage patients to also explore further aggressive treatment options such as ReRT. Thus it remains uncertain what proportional impact the ReRT provides in extending survival duration compared to that provided by BEV alone or simply having favourable clinical factors. Although the study outcome is hypothesis generating, these issues need to be considered when appraising the potential benefits of aggressive interventions at time of chemorefractory relapse.

## Conclusion

A management policy in this clinic for selected patients with recurrent GBM that reserved BEV until symptomatic chemorefractory disease provided an improvement in median survival compared to best supportive care. This strategy especially when combined with ReRT may provide a meaningful increase in survival duration.

## Abbreviations

BEV: Bevacizumab
BEVend: Last bevacizumab cycle
BEVreject: Decision to decline bevacizumab
BEVstart: Decision to start bevacizumab
BSC: Best supportive care
GBM: Glioblastoma
PFS: Progression-free survival
RANO: Revised Assessment in Neuro-Oncology
ReRT: Re-irradiation
VEGF: Vascular endothelial growth factor
